# Alcohol and breast cancer risk: Middle-aged women’s logic and recommendations for reducing consumption in Australia

**DOI:** 10.1371/journal.pone.0211293

**Published:** 2019-02-13

**Authors:** Samantha B. Meyer, Kristen Foley, Ian Olver, Paul R. Ward, Darlene McNaughton, Lillian Mwanri, Emma R. Miller

**Affiliations:** 1 School of Public Health and Health Systems, University of Waterloo, Waterloo, Ontario, Canada; 2 College of Medicine and Public Health, Flinders University, Adelaide, South Australia, Australia; 3 University of South Australia Cancer Research Institute, Adelaide, South Australia, Australia; Northumbria University, UNITED KINGDOM

## Abstract

**Background:**

We aimed to understand the factors shaping alcohol consumption patterns in middle-aged women (45–64), and to identify participant-driven population- and policy-level strategies that may be used to addresses alcohol consumption and reduce breast cancer risk.

**Methods:**

Semi-structured interviews (n = 35) were conducted with ‘middle-aged’ women conversant in English and living in South Australia with no history of breast cancer diagnosis. Data were deductively coded using a co-developed framework including variables relevant to our study objectives. Women were asked about their current level of awareness of the association between alcohol and breast cancer risk, and their personal recommendations for how to decrease consumption in middle-aged Australian women.

**Results:**

Women discussed their previous efforts to decrease consumption, which we drew on to identify preliminary recommendations for consumption reduction. We identified a low level of awareness of alcohol and breast cancer risk, and confusion related to alcohol as a risk for breast cancer, but not *always* causing breast cancer. Participants suggested that education and awareness, through various means, may help to reduce consumption.

**Conclusions:**

Participants’ description of strategies used to reduce their own consumption lead us to suggest that campaigns might focus on the more salient and immediate effects of alcohol (e.g. on physical appearance and mental health) rather than longer-term consequences. Critical considerations for messaging include addressing the personal, physical and social pleasures that alcohol provides, and how these may differ across socio-demographics.

## Background

### Alcohol and breast cancer risk

Globally, alcohol is identified as causing significant disability, disease and mortality [1] and is considered a risk factor for a range of soft tissue cancers [2, 3] including breast cancer [4, 5]. International research suggests that consumption at low levels previously considered ‘safe’ significantly elevate cancer risk [4–7]. Cancer Council Australia recommends that people limit their alcohol, or avoid alcohol all together [8]. However, the regular consumption of alcohol is widespread in Australia—roughly 17% of Australians use alcohol at levels that put them at risk of harm over their lifetime [9]. These statistics are similar to high risk consumption rates seen in other developed countries, reinforcing the global nature of this public health problem. For example, in 2016 19.0% of Canadians aged 12+ reported alcohol consumption that classified them as heavy drinkers [10]. Similarly, data from 2015 suggests that 26.9% of Americans aged 18+ reported that they engaged in binge drinking (~4 drinks for women and 5 drinks for men in 2 hours) [11].

The consumption of alcohol—how, what, when, where and in what ways it is consumed—is shaped by a complex system of social, psychological, behavioural, economic, legal and physical environmental factors that interoperate at individual, community and population levels [12]. Although there has been some success in reducing rates of consumption in Australia (a decrease from 20% in 2010 to 17% 2014), reducing consumption levels to meet national guidelines remains a challenge [13] as alcohol consumption in Australia has been described as ‘culturally ingrained’ [14]. Notably, alcohol consumption levels among ‘middle-aged’ (45–64 years) Australian women remain higher than among other age groups [15–17]. In this same group we have also witnessed the trebling of cancer incidence over the past 50 years [6]. Thus, alcohol remains a notable public health problem [18] with the attributable risk of alcohol for all cancer being ~6% [8].

In order to minimise breast cancer risk, it is critical that women understand the risks associated with alcohol so they can make informed decisions regarding consumption [19, 20]. According to an Australian Institute of Health and Welfare (2014) report, the awareness of breast cancer is high in Australia, relative to other cancers [21]. However, public knowledge regarding the association between alcohol consumption specifically and cancer risk remains poor [13, 22, 23]. Just under half (47%) of Australians identify drinking alcohol as a risk factor for cancer [24]. Furthermore, knowledge about alcohol as a risk factor for cancer may be poorest among the heaviest drinkers in Australia [24]. This is concerning, given new evidence suggesting a dose-response-relationship between alcohol and breast cancer. That is, the greater the quantity of alcohol consumed, the greater the risk of breast cancer. The excess risk for cancer among women who drink heavily is estimated to be 60% compared to non-drinkers [25]. Clear and meaningful communication of the potential role of alcohol in cancer causation, and the promotion of behaviour change strategies for modifiable risk factors, are central to prevention [26] and to decreasing cancer incidence [6].

### Current interventions to reduce alcohol consumption in Australia

To date, extensive research in Australia and abroad has looked at interventions to reduce harmful drinking. In particular, much of the effort to minimise risky alcohol consumption has focused on developing and implementing preventive interventions in primary health care settings [27–30]. Despite considerable evidence for the efficacy and cost-effectiveness of such primary health care-based approaches [31–36], widespread incorporation of alcohol prevention in routine care has not occurred in Australia or elsewhere [31, 36]. Furthermore, widespread incorporation of interventions in relation to alcohol-related cancer prevention specifically is unlikely given the evidence that only 37% of general practitioners attending health prevention seminars around Australia agreed that the regular intake of alcohol, even at light levels, can lead to cancer [37]. Specific to breast cancer risk, much of the intervention literature focuses on diet (not specific to alcohol), physical inactivity [38–40], and improving mammography screening uptake [41].

### Study rationale and objective

Existing literature suggests that there is a need for greater training for primary care providers regarding alcohol and cancer risk, communication strategies for use in primary care settings, and the development of interventions outside of the primary care setting [42]. However, to date the research necessary to inform population- and policy-level interventions is limited. In response, the present study sought to identify women’s perceptions of the role of alcohol in breast cancer risk and investigate the complexity of their logic with regards to alcohol consumption and reduction. Critical reflection of women’s logic around consumption provides foundational information to begin to establish participant-driven population- and policy-level interventions that may be used to addresses alcohol consumption, and either directly or indirectly reduce breast cancer risk. Our work also furthers conversations regarding the complexity of interventions required to reduce alcohol consumption in this population.

## Methods

Qualitative interviews were conducted with middle-aged women (45–64) conversant in English and living in South Australia with no history of breast cancer diagnosis in order to identify how to decrease alcohol consumption in middle-aged Australian women. We asked women about their current level of awareness of the association between alcohol and breast cancer risk, and their personal recommendations for how to decrease consumption in Australia. Their accounts of risk perception allowed us to identify current perceptions of alcohol in this population group, and to better understand current consumption patterns, specific to this age group. Women discussed previous efforts to decrease consumption, which we were able to draw on to identify preliminary recommendations for consumption reduction. Finally, we asked women what they thought government and health organisations could do to address alcohol as a risk factor for cancer in Australia. The complete interview schedule can be found in S1 Table.

Women were purposively sampled to ensure diversity with regards to perceived susceptibility to breast cancer, perceived level of alcohol consumption, and socio-economic status (perceived relative income and education). These factors have been identified in the literature as related to knowledge of breast cancer risk [43–45]. Our study was advertised via community paper advertisements, Facebook, organisational email lists/e-bulletins, and via newspaper and local radio interviews. Interested women were asked to complete a two-minute survey (online or via telephone) to identify their age, education level, income, general health, perceived risk of breast cancer, and perceived level of alcohol consumption. A selection hierarchy was developed to form 12 possible risk-socio-economic-alcohol subgroups (Table 1). A total of 122 women were recruited to complete a survey. Based on their responses, we identified a sample and conducted semi-structured interviews with one to three women belonging to each of the potential 12 groups, with 35 women interviewed in total (4 were pilot interviews but have been included in the overall sample). No repeat interviews were completed.

**Table 1:** Stratification of purposive sampling procedures (n = 126).

**Perceived susceptibility to breast cancer (n)**											
Likely (18)				Neither likely/ unlikely (30)				Unlikely (78)			
**Perceived level of alcohol consumption (n)**											
None/light (7)		Moderate/ heavy (11)		None/light (17)		Moderate/ heavy (13)		None/light (35)		Moderate/ heavy (43)	
**SE score [education level plus perceived relative income] (n)**											
> median (4)	≤ median (3)	> median (7)	≤ median (4)	> median (10)	≤ median (7)	> median (8)	≤ median (5)	> median (19)	≤ median (16)	> median (23)	≤ median (20)

For the purpose of the present paper, data are not analysed according to subgroups, though we acknowledge that the group of women interviewed are not homogeneous. Given the role of risk perception and socio-economic status in alcohol consumption, we feel it critical to explore how these characteristics shape, or are shaped by their personal experiences in greater depth, beyond the objective of the present report. As such, a subgroup analysis will be the subject of another report.

Women were contacted by phone to organise a time for the interview (no relationship was established with women prior to commencement of the interviews). Participants were informed during this call that we wanted to understand what women in their age range think causes breast cancer and also the place of alcohol in their social lives. This information was included in the recruitment material. Of the women we contacted one did not respond to phone messages and one withdrew after scheduling an interview, stating they had changed their mind about participating. Researcher KF (female) conducted the semi-structured interviews. Participants were informed that KF was a research assistant, who has training as an Occupational Therapist and also in Public Health Research and has experience with qualitative research projects. KF’s prior research experience was not in the area of alcohol or alcohol-related cancer. That she was younger than the age group of interest may have reduced the potential for experiential assumptions. It was important for KF to reflect on the pervasive social place of alcohol in the community in which the study was conducted, which is likely to have shaped the perspectives of both interviewer and interviewee alike.

Interviews took place in participant homes, cafes, community libraries and civic spaces and averaged approximately 45 minutes in duration. A reflective journal was used to record field notes. No one else was present during the interviews, although in some settings there were passers-by. All women received a $30 shopping voucher as compensation for their time. Audio-recordings were transcribed verbatim by a professional transcriptionist in South Australia who signed a confidentiality agreement. We followed a framework analysis approach (see [46]). Building on an a priori framework of barriers/facilitators to reducing alcohol consumption and factors supporting/inhibiting messages about breast cancer risk (developed from the literature), inductive coding on three transcripts was conducted by three members of the research team independently to establish a coding framework. The subsequent transcripts were deductively coded to this framework by KF, with new codes added throughout. Throughout this process the coding was presented back to the research team to ensure agreement in the analysis and presentation of data. Data were managed using NVivo version 11 [47]. Data saturation was discussed by the team to have been reached when no new codes emerged.

This study was given ethics approval by the Flinders University Social and Behavioural Research Ethics Committee (Project #7479). All participants provided written or verbal consent for participation. Pseudonyms are used in the reporting of results. Participants were not involved in reviewing their transcripts nor in data analysis and interpretation.

## Results

Prior to presenting strategies suggested by women to reduce alcohol consumption, it is important to contextualise their recommendations with data regarding their existing knowledge about alcohol as a risk factor and their logic regarding their own alcohol consumption. As we identify, their current level of knowledge laid the foundation for their alcohol-reduction recommendations.

### Existing knowledge about breast cancer risk

#### Awareness of alcohol as a risk factor for breast cancer

The majority of women we interviewed were not aware that alcohol is a risk factor for breast cancer. They were more likely to speak of risks related to age, lifestyle (an unhealthy diet and inactivity), genetics, and stress. Women were more likely to speak of the harms of alcohol for other medical conditions, for example, liver disease or leading to harm if consumed during pregnancy. When prompted to consider alcohol as a risk factor for breast cancer specifically, women spoke of risk as being related to frequency and quantity of consumption—i.e. the more often and the more you drink, the greater your risk for breast cancer. The type of alcohol consumed—for example, spirits vs. wine—was also considered to affect risk level.

“I think the more you drink, it’s more likely. And, I wonder…whether more of the spirit-type heavy drinks may be related? … But just a thought, but, certainly the more you drink, yeah, definitely, would be more common, I think. Yeah the type of drinking might be related too.”

(Malorie)

Related to the dose-response perception, participants had difficulty defining what was ‘too much’ and what is considered a ‘safe’ level for consumption.

“I don’t even know why but I’m thinking like five drinks. I don’t even know why but like… Yeah, more than five drinks a week I think is—and I suppose like alcohol content and whatever can vary but I don’t know if I’ve read something or if I’ve just made that up, or whatever, but I think that consistently doing that kind of thing can be—I’m not saying if you do it like once or twice but, you know, I’m thinking consistently, if it was like an every week thing, like years on end, I’m sure that would be a factor.”

(Toni)

Also related to dose-response, women identified ‘binge’ drinkers as individuals who they perceived to be at risk, or ‘candidates’, for breast cancer as a consequence of their alcohol consumption. For example:

“Binge drinkers or people who drink every day, heavily every day. That’s got to have some effect on the body, some negative effect; it can’t be good.”

(Lauren)

#### Alcohol as a risk factor is logical as it is generally unhealthy

Despite not knowing about alcohol as a risk factor for breast cancer, women identified the association as a ‘logical’ one given the known status of alcohol as being negative for health more generally.

“Yeah, so it must be a lot of information…about alcohol really and breast cancer so I kind of didn’t think ‘oh wow, that’s weird, they’re putting those two together’—I don’t know why but like I just see alcohol as that kind of factor behind things.”

(Toni)

Only one participant, after being told of the association, did not believe that alcohol consumption was a risk factor in breast cancer.

“I don’t believe that drinking alcohol causes breast cancer at all, no matter how much alcohol you drink. You could drink three litres a day and I don’t think that would cause breast cancer. It may cause having no liver but—or even liver cancer, maybe, but not breast cancer.”

(Trudy)

Her dismissal was founded upon her personal experience—the fact that her own personal alcohol consumption had not led to breast cancer. Though no other participant was as explicit in their disbelief, personal experience led many others to at first question the role of alcohol in breast cancer.

“Well, my good friend that I mentioned earlier [who was diagnosed with breast cancer], and I know for a fact that she doesn’t drink a lot of alcohol…Another lady that I know of, I don’t know her very well but I wouldn’t suspect she would be a big drinker either. She actually got breast cancer—and like these two ladies are a similar age to me—she got breast cancer probably about ten years ago so she would’ve been in her 40s.”

(Kennedy)

“…my mother was an extreme healthy person, so she didn’t, not a drop of alcohol passed her lips. She, like I said, ‘chocolate is poison’. She was very good, very slim, fit, healthy, until she got cancer. And then, she died. But, you know, took a long time, but you go, ‘well, if it’s going to happen to someone like that, then…’, and I know, that’s illogical, but for me I go, ‘well, she didn’t drink, and look what happened to her’. So, that sounds pathetic I know, but it’s what I do.”

(Theresa)

Participants referenced friends or family who do not drink, or do not drink quantities that might be identified as unsafe, but were diagnosed with breast cancer nonetheless.

#### Breast cancer as survivable and common

Breast cancer was commonly described as ‘survivable’ and ‘treatable’.

“it just seems like there’s a pretty good survival rate these days if it is, I suppose, just breast …”

(Kennedy)

“It is one of the most curable forms of cancer if it can be caught early.”

(Margaret)

“If you do have breast cancer you can have your breast removed and you can have chemotherapy, radio treatment, but I have read that the survival rate for breast cancer is a lot, lot better now than it ever has been in the past.”

(Sarah)

Breast cancer was also identified by some women as being very common, which may impact their perceived importance of modifiable risk factors.

“…so many different women seem to get it and at different ages and, yeah, it just seems to me to be the most prolific form of cancer that women get.”

(Kennedy)

The logic that ‘everyone gets breast cancer’ and that breast cancer risk is beyond individual control was prominent throughout. While not explicitly stated, the data suggest that low perceived risk of a breast cancer diagnosis may be used as a justification for engaging in high risk behaviours, such as consuming alcohol.

### Recommendations for how health organisations and government can decrease consumption

#### Improving education and awareness

The need for education and awareness was raised, both prompted and unprompted, during discussions regarding how to reduce alcohol consumption at a population level. Education refers to the process of imparting knowledge, and the tools (e.g. numeracy and literacy) to use that knowledge to make an informed decision regarding alcohol consumption. Raising awareness, often achieved through education, is about changing the perception of the risk of alcohol as it relates to breast cancer.

Some participants said that in order for the messaging about alcohol consumption and breast cancer risk to be received by the public, health organisations and government need to provide the public with evidence of the association.

“…if someone came up to me and said ‘even that one glass of wine that you have three or four times a week, or whatever, is really increasing it by 50 percent or 30 percent’ I’d be like ‘oh’. That would make me stop and think a little but then I’d want some really hard evidence of that because really …the medical world changes their opinion on what is beneficial and what the cut-off point is before it becomes a worry, like coffee consumption, like alcohol consumption, like chocolate consumption.”

(Angela)

“If they said they found that, you know, out of a group of, you know, your standard, you know, so many hundred that 50 percent ended up with cancer who drank over a certain amount then I’d start thinking ‘oh, goodness, that is really’—you know, they just say it’s a contributing factor but they haven’t really given, you know, real strong statistics.”

(Josephine)

Valerie noted that there is “a lot of confusion around information. There’s so much information it’s like it’s almost impossible to—it’s a minefield.” As a consequence, she noted the importance of giving “more straight up information…about the physiology of what it actually does in your body and if you have one and then maybe five, what the difference is there, then across time.” She also discussed the urgency at which people want health information, and that organisations need to make it easy to access the information quickly.

Visual depictions of the consequences of alcohol were identified as being something that might help to make the association more salient.

“Or maybe if you had an advertising campaign that showed the dangers of drinking alcohol and what it can do to a mature woman’s body, the shock value might make a difference because I just don’t associate the two and I’m not sure how many other women would…because we know better, it’s just you don’t think about it.”

(Lauren)

Participants also spoke of the role of media education, specifically drawing on the example of smoking. Education was described as providing facts and not just telling people what to do—the central point being that education and facts provide a means for making a better choice, but a choice nonetheless.

“…education in alcohol is the way to go rather than ‘you can’t do this anymore’ because the minute people think that someone’s telling them what to do they don’t like it whereas if you’re just being given facts it’s a different story…You’re given the facts and—yeah, I just think education works because if you’re completely informed you can then make a better choice. If you are just told you can’t do that you don’t like it.”

(Paulette)

Given the limited awareness of the link between alcohol and breast cancer, it is unsurprising that many women spoke of the importance of raising awareness as a step in reducing consumption.

“Well, I think we need to do it better. If you guys are seeing a link I think we have to get that message out there …I think you’ll have to get the message across about the alcohol but I think that there is already, compared to the other cancers, a lot more support.”

(Marcy)

As a consequence of poor awareness, Sabrina identified the long road ahead to making the association known: “I think it’s going to take a time for the alcohol message to get through. I don’t think a lot of women would link alcohol with increasing cancer. I don’t think a lot would naturally think that way, so I think that’s a way.” (Sabrina)

#### Campaigns targeting behaviour change

Many women made reference to existing smoking campaigns and their success, as well as other targeted cancers that have become commonplace in Australia.

“Well, they’d have to copy the other campaigns… I mean why has the Slip, Slap, Slop campaign been so successful? Why are we wearing hats in Australia? It’s been a very successful campaign. Why are we going for pap smears? Why are we doing the bowel test that the government sends us when we’re over 50? Why do I go for my mammogram? Because the government says ‘you’re 50, go for your mammogram’ those campaigns. I think this is a first world country, we can do it very successfully, like we’ve done other things.”

(Marcy)

It was suggested that a similar tactic—using images that identify the harms of alcohol—might be effective for the reduction of alcohol consumption.

“… Maybe we have those graphic photos of ‘this is what alcohol can do to you’ because, as you said it to me, my first thought was the gross images so maybe that’s—yeah.”

(Tara)

“The education of seeing women staggering everywhere and—you know, in mini-skirts and…I’m laughing but it’s not funny. Like it’s not funny. I’m laughing saying it but it’s not funny … I just think ‘oh you just look terrible. You just look disgusting’.”

(Paulette)

While Tara identified images related to the impact of alcohol on health, Paulette spoke about the social risks of alcohol as a strategy for reducing consumption. Related to social acceptability, Toni and Jenna’s comments also reflect how social perceptions of alcohol consumption (at various levels) may affect behaviour.

“I think if there’s a campaign around, you know, educating women, you know, what is acceptable alcohol consumption and what isn’t acceptable or what maybe is a bit of a red flag.”

(Toni)

“…You know, but, yeah I mean it’s really embarrassing, especially as a teacher, ex-teacher, to be picked up for drink driving. Well, one it’s not safe, and two, it’s just plain embarrassing, you know you’ve got your reputation to look after.”

(Jenna)

The above comments are indicative of the role that shame or embarrassment may play in shaping alcohol consumption.

References to hangovers, feeling sluggish, and the impact of alcohol on appearance were all factors that led women to decrease consumption or abstain from drinking altogether.

“Like even that bottle shared with the girlfriend the other night, it was kind of like too much for me really at this stage, so I could feel it—although I exercised yesterday…I can feel it still in my body, put it that way.”

(Valerie)

“I don’t metabolise wine very well so it makes my brain fuzzy so I don’t drink at all if I need to concentrate on anything.”

(Lia)

Caitlin referred to how alcohol consumption impacts her mood and consequently affected her behaviour towards her children, leading her to reduce consumption.

“…I would always be the most chilled mother when I was at the height of that two or three glass thing but then all of a sudden it’d go down and I’d get really cranky and really impatient with them because that always was about bedtime that I’d start coming down and I’d get really annoyed with every little thing and that was another reason why I did it.”

(Caitlin)

The effects of alcohol on appearance and specifically weight was identified as problematic and led women to reduce the quantity of alcohol consumed.

“I think that if you messaged women about how many calories are in certain wines and beers and what have you it’d be like ‘oh shit’. I think women think more about weight as well so I think that could be effective, to go ‘oh, holy crap. I’ve just drunk that bottle of wine and that was that many calories and that many calories happens to be an extra day’s eating or something’.

(Sabrina)

“But when you’re younger, you can, you don’t gain the weight, and so when you’re older, whether it’s food or whether it’s alcohol, or, or food intake, you do gain the weight.”

(Malorie)

The ‘next-day’ impacts of drinking were identified as a cause for not drinking at all, or reducing consumption.

“Also the next day be able to go out for a walk or go to the gym or do whatever I need to do without feeling under the weather.”

(Toni)

“I don’t drink much now and it’s all just like not wanting to deal with the consequences, not wanting a hangover. Not wanting to have my body go through the processing of a lot of alcohol, but I still enjoy a drink.”

(Valerie)

The after-effects of consumption were noted as a problem that was more difficult for women as they aged.

“…when you’re younger you kind of like feel this I think infallibility and the—I don’t think the alcohol affects—it obviously affects you but the feeling of the effect I don’t think is as extreme. As you get older the feeling of it is just like ‘oh my God’ you know, you feel like you’re going to die.

(Valerie)

“…As I get older I think, you know, I’ve just sort of learnt what you can and can’t get away with and certainly recovery as you get older is a lot harder, recovery from the hangover.”

(Tara)

The physical effects of hangovers were consistently noted, as were the consequences of feeling ill and perceptions that age makes it harder to recover. Women noted the difficulties that hangovers meant in terms of productivity, and feeling like a day after having a drink was a ‘lost day’.

“…I get sick of waking up and losing a day… and so there’ll be a point where you think ‘yeah, it’s great. I can drink forever tonight. I can keep going. I can keep going’ but somewhere I will start to go ‘I’m going to have water. I can’t keep doing this’ and if I do I’m not well and it’s horrible and we all know that.”

(Louise)

“If you drink too much every night, especially on the weekends, you wake up the next day feeling—you waste your weekend. I think that was the first thing that really started to get to me. If I drank too much on a Friday, Saturday night all day Saturday and all day Sunday were stuffed… I find I don’t want to stay up until two or three o’clock in the morning drinking, I want to get up in the morning and do stuff in the day time.”

(Rachel)

Lauren and Josephine made specific reference to the fact that it was not breast cancer that worried them about consumption but rather, the impact on memory.

“I try and keep it [alcohol consumption] down though … I’m not so much worried about breast cancer… not in the slightest… I guess that’s more of what I worry about, memory and stuff, because sometimes I think I forget what I do from one minute to the next.”

(Josephine)

“I was noticing some changes with my memory and that really sparked—that really scared me… I’d ask my daughter ‘what are we having for tea?’ She’d tell me and then an hour later I’d ask the same question; wouldn’t remember that I’d said it.”

(Lauren)

In Lauren’s case, the impact on memory served as an impetus for reducing alcohol consumption.

Women were asked what they would suggest if a friend came to them for advice on how to reduce their alcohol consumption. The aim of this question was to identify strategies, developed by participations, for reducing consumption. Strategies provided were related to making substitutions—for example, swapping alcohol for different types of beverages.

“…switch to low alcohol products. See in the pub often I’ll have a spacer of a water between drinks, that sort of thing, and you know, they know what their temptations are.”

(Sabrina)

Some women referred to strategies to occupy their time that do not include alcohol. For example:

“get your mind off it by doing something else, finding new hobbies or something like that maybe.”

(Angela)

“Distraction. That’s how I gave up smoking years ago, I cleaned the house every five minutes because…–it was a distraction so that would be the same with any vice. If you want to slow it down or get rid of it do something else…Yeah or change your routine, change your habits.

(Trudy)

Participant responses suggest that distracting oneself, or finding alternative activities may be strategies for changing consumption patterns.

### What can be done at the policy/legislative level?

When asked about what the government or health organisations should do to reduce consumption, women also referred to what we identify as policy or legislative changes. For example, labelling alcohol products was provided as a strategy for reducing consumption.

“I know this because of being in the industry but the education on the bottle saying how many standard drinks are in there. I know depending on the alcohol volume the standard drinks change so therefore I look at it and go ‘oh, okay, this one’s a bit stronger so I probably shouldn’t have quite so much’.”

(Paulette)

The existing legislation regarding alcohol consumption and driving was often mentioned as a factor that influenced consumption patterns. Driving was identified as a socially acceptable rationale for not drinking, and gave women an ‘out’, avoiding peer pressure.

“…like Saturday night when I’m going out with friends I won’t have two drinks because I’ll have to drive home. I think that’s a lot more acceptable these days too. People are a lot more wary of not being over the limit when you’re driving and I think that’s education as well.”

(Nicole)

“No. If I’m driving I wouldn’t drink anyway because I know that for me a lot less than the legal limit will affect my reactions a lot so I’m not confident with any kind of alcohol if I’m doing something like that.”

(Lia)

Taxation was identified by some participants as a proposed strategy for decreasing consumption.

“For me, for it to be effective you’d just crank up the price. I think it was earlier this week somebody was suggesting putting a higher tax on sugar drinks, like soft drinks and stuff, and apparently Malcolm Turnbull’s said ‘no, we pay enough taxes anyway’. I think that would be an incentive—it would for me—but, you know, unfortunately I think the people that can least afford it are the ones that would still pay for it on the whole, across the population.”

(Nicole)

However, others felt that taxation as an approach to reduction would be ineffective. Explanations for the failure of such an approach included people continuing to find a way to purchase alcohol because it is something they enjoy. The pleasure, both physically and socially, of drinking was expressed as follows:

“…even if there’s a government incentive or government tax about that I’m still going to probably be indulging because I enjoy it or I like it or I like the taste of it or it’s just a habit, or all of them.”

(Toni)

“Perhaps like taxing—no, that’s not going to work because it doesn’t work for people that smoke so it’s not going to work for people that really like to drink.”

(Angela)

Cynthia suggested that strategies targeting different sub-populations are required. She indicated that her income and her education make her unique, and that certain strategies might work for some population groups, but not others.

“…You know, giving people information is not going to change their behaviour. I’m well educated. I have all the information. I know the risks…. We’re middle class people; we know stuff….You’ve got all that sort of methodology over here and you’ve got your behaviours and your information, so making, you know, settings, putting tax on alcohol, you know, that wouldn’t affect me because I have the means to overcome the tax whereas tobacco, you know, now an average cost of …a packet [of cigarettes] has gone up to $35, a huge incentive to stop smoking, whereas the way we structure our taxes on alcohol, that isn’t going to affect somebody like me. It might affect a pensioner. It might affect a woman who’s stopped working but it’s not going to affect me and it’s not going to affect most of my friends.”

(Cynthia)

Josephine suggested that there have been changes over time in terms of the availability of alcohol that may play a role in increased consumption.

“…well, yeah, so you drive past them all the time whereas years ago you had to go out of your way to get it.”

(Josephine)

She noted the number of locations where you can purchase alcohol and how this is likely an influence on consumption. She suggested that accessibility is an issue the government might address to reduce consumption.

### Decreasing consumption is the responsibility of the individual

Alcohol consumption was identified as a personal issue by some participants, leading them to conclude that reducing consumption is not the responsibility of health organisations or government. Behaviour change for these women related to willpower, resilience, initiative and taking control. This was said to require self-reflection to first identify the problem and then take action.

“Well, you know, I was—I just thought ‘this is not sensible behaviour. This is unhealthy behaviour. You don’t need to be doing this. There are probably some risks here for your health’”

(Cynthia)

Despite seeing it as an individual responsibility, the strategies used by women, discussed below, may be used to inform messaging targeting behaviour change at a population level. Women talked about methods for cutting back or cutting out alcohol all together, and the strategies used to do so. Self-monitoring, rule setting, writing down their consumption, or using tools to measure how much they are drinking were methods identified as helpful in controlling alcohol consumption. For example:

“That’s one way and the three alcohol free days just to say ‘no, not going to drink on these days; that’s it’.”

(Sophie)

“… I started marking on the calendar. I had a [name of] changing my drinking behaviour and I decided that I had to have more non-drinking days in a month than drinking days so I had to have at least 15 non-drinking days and that should bring me down. I tried to think about not having, you know, more than this and more than that, but what I in the end did was count drinks over a week …”

(Cynthia)

“I would like to buy a set of glasses that have got the line because it would be more of a mental trigger to go ‘okay, no, that’—you know, from time to time I think about exercising willpower, measuring out 125ml but if the line’s not there, well, you know, you can…”

(Carrie)

Changing consumption patterns was another method identified for reducing alcohol intake—for example, purchasing smaller quantities, switching to lower alcohol content beverages and removing stocks of alcohol in the home.

## Discussion

### Alcohol and breast cancer: Changing risk perception

The level of awareness of alcohol as a risk factor for breast cancer was low in our group of participants. Participants initially questioned the association based on their knowledge of people who drink and do not have cancer, or who do not drink and do have cancer. The confusion related to alcohol as a risk factor for cancer, but not *always* causing cancer. These data are in line with research identifying that individuals engage with multiple and often conflicting frameworks when making risk-based decisions [26]. Individuals’ perceptions of risk, or whether they or others can be regarded as a ‘disease candidate’ [48, 49] are reliant on the input of personal, social and cultural sources as well as expert-endorsed or celebrity-endorsed information. Our participants drew on their personal experiences to build an evidence-base that was seen as incongruent or conflicting with the information we provided regarding alcohol as a risk factor. While this demonstrates the challenges that might be faced in communicating the risks of alcohol at a population level, our data also suggest that the association might not be such a hard sell. The women reported widespread knowledge regarding alcohol having a negative impact on health more generally. As such, it ‘made sense’ or was seen as ‘logical’ that it would be associated with breast cancer. These data suggest that changing risk perception and the notion of what a breast cancer ‘candidate’ looks like, may be obtainable.

### Reducing consumption requires more than education and awareness

The lack of awareness of alcohol as a risk factor for breast cancer may suggest that the first step to reducing consumption in this group of women is education and awareness. Inconsistent with previous Australian research [50], our participants supported the notion that providing women with more information about the association might help reduce consumption. However, while effective information about breast cancer risk has been argued to help women to make more accurate calculations of their personal risk [19], data are not convincing regarding the role of messaging in reducing consumption. Australian campaigns targeting alcohol behaviour via mass media have had limited success; likely a consequence of the overshadowing of persistent product marketing, social norms, and behaviours driven by addiction or habit [51]. Indeed, Dixon, Pratt (13) argue that while campaigns can raise awareness regarding the links between alcohol and cancer, as well as knowledge of drinking guidelines, “a single campaign may be insufficient to measurably curb drinking behaviour in a culture where pro-alcohol social norms and product marketing are pervasive” (p. 1).

Participants spoke of the success of smoking and skin cancer campaigns, and suggested that the government and health organisations should consider following similar lines of messaging to reduce alcohol consumption. However, the success of campaigns in Australia targeting smoking and skin cancer required extensive investment—both time and monetary. Alcohol is significantly ‘ingrained’ [14] in the Australian culture, and so it is likely to take decades before we see similar effects to what have been witnessed with campaigns on sun safety and smoking reduction.

### The importance of taking into account social norms and habits

Exploring women’s logic regarding consumption provided us with insight regarding the social norms and habits that shape their behaviours. Their social norms and habits were particularly salient when women spoke of how and why they have made changes in their lives to reduce their alcohol consumption. Women spoke of choosing lower alcohol content beverages, switching to non-alcoholic beverages, choosing to be the designated driver, and self-monitoring their intake until lesser quantities became habit. Their rationale for changing habits provides a window into the values of these women, and what they may prioritise over drinking, providing opportunity for their voices to be acknowledged in campaign messaging. They identified the negative effects of alcohol in their lives; namely the impact on their weight, overall mood, social relationships, sleep, and productivity. It is possible that providing targeted messaging regarding how a reduction in alcohol consumption may improve their social, physical and mental health will be a way to indirectly lower breast cancer risk. However, we acknowledge that providing messages of this nature, while appealing to the cognitive evaluative processes, do not address the affective processes that shape risk perception [52]. As noted, individuals engage and consult with multiple, often conflicting frameworks when making risk-based decisions [26]. The data do however suggest that perhaps education about the health risks of alcohol will not be as persuasive as an emphasis on the more salient and immediate effects of alcohol.

### The role of industry in alcohol consumption

In addition to the social norms and habits shaping consumption, we acknowledge that the alcohol industry (product marketing) and competing messages [53] likely play a larger role in shaping consumption than is identified in our data. Research demonstrates that Australian adults are regularly exposed to advertisements that depict alcohol consumption as fun, social and inexpensive, reinforcing existing cultural norms that prevent them from meeting current consumption guidelines [54]. In fact, the alcohol industry have been identified as actively working against health messages and purposefully working to embed alcohol into Australian culture [55]. Therefore, to achieve reductions in alcohol consumption, it has been recommended that continual education regarding the harms of alcohol needs to occur within a range of national, state and local policy controls. These controls may include managing the physical availability (access) and economic availability (price) of alcohol, and addressing the cultural place of alcohol in Australia through targeted and research-based social marketing and public education platforms, which have been recommended elsewhere [13, 42, 56]. However, Australia is a climate arguably weighted towards the promotion of alcohol, playing a large force in what can and will be done at a policy-level to reduce alcohol consumption. Industry, as a form of revenue, play a large role in what happens at a policy-level. Indeed Bates, Holmes (22) argue that changes to policy controls need to be considered within the context of the revenue generated by the alcohol industry in Australia, as well as citizen concerns for individual freedoms.

While our participants did not refer to the role of industry or government in the promotion of alcohol, nor the impact this may have on their alcohol consumption, there was an overall sense of support for government interventions targeting alcohol consumption (e.g. labelling and taxation). Public support for alcohol-related policies has been found to be associated with the extent to which policy are deemed intrusive or restrictive, with people tending to prefer policies they perceive to impact other people and not themselves [22]. The women in our study, for the most part, were supportive of reducing their alcohol intake by their own accord, suggesting that such policies would not have a significant impact on their lives. This likely explains their support for government interventions.

### Risk perceptions surrounding breast cancer

Our data offer novel insight regarding the role that risk perception, as related to a breast cancer diagnosis, may play in women’s alcohol consumption. Within our discussions, women spoke of breast cancer as a ‘survivable cancer’. This may influence the extent to which they regard messaging about the reduction or elimination of behaviours they enjoy, such as consuming alcohol. In a similar vein, some women identified breast cancer as being extremely prevalent—the notion that ‘everyone gets breast cancer’. We question whether efforts to reduce risk may be impacted by this perception, with women assuming that behaviour has little to do with whether they will be diagnosed. The notion of optimistic bias, or overly optimistic judgements about breast cancer risk [57] may be helpful in explaining some women’s engagement in alcohol consumption despite known risks. Empirical research suggests that women often perceive their personal risk of breast cancer to be exceedingly less than that of other women. The low perception of risk has been found to have negative implications for engagement in preventative measures [58], and therefore may influence willingness to make lifestyle changes, including reducing alcohol consumption.

### Strengths and limitations

This research provides an in-depth understanding of how women in a high-risk age group for breast cancer view the barriers/facilitators to reducing alcohol consumption as a modifiable risk factor for breast cancer, as well as factors supporting/inhibiting messages about breast cancer risk. The semi-structured interview method enabled the direction of inquiry to be guided by the participant as well as the key areas of interest. This participant perspective will support policy makers and social scientists in understanding more about the processes of risk communication. The research was undertaken by a multidisciplinary team comprising epidemiology, sociology, oncology, anthropology and occupational therapy. This supported trustworthiness during analysis and interpretation of the data as team members were able to interrogate the findings from a variety of professional dispositions. Further, the framework analysis method [46] provided a systematic approach with which to explore information central to our research objective.

Another strength of this study was the diversity of women we interviewed according to self-perceived alcohol consumption, breast cancer risk and socioeconomic status. A limitation in this respect, however, was that we sampled by self-reported diversity. This was undertaken in this way to help examine how women’s perceptions of breast cancer risk and alcohol consumption levels influenced their attitudes about alcohol consumption. This strategy, however, means we do not have an empirical picture of the differences in socio-demographic, risk status for breast cancer and level of alcohol consumption. Similarly, the self-selecting nature of our sample may have been a limitation to the research. Because the research was undertaken only in South Australia the results may not be generalisable to other contexts. It is also important to acknowledge that our data analysis does not take into account diversity across middle-aged women, and the extent to which individual characteristics and experiences will shape intervention effectiveness, or lack thereof. Further, we do not explore the role of gender in shaping consumption patterns, and how findings may differ in men of this age group. However, our data and critical reflection allow us to propose considerations in the development of risk communication that can be tested through other research methodologies.

## Conclusions

As new evidence comes to light supporting the link between alcohol and breast cancer, more needs to be done to shift consumption in middle-aged women whose intake remains higher than among other age groups [15–17]. Our participants suggest that the provision of convincing evidence may help to change consumption patterns in this age group. Campaigns to reduce consumption may also consider building messages around the more salient and immediate effects of alcohol identified by our participants, namely physical appearance and mental health. However, it will be critical that in the development of messaging, health promotors consider how to address the personal, physical and social pleasures that alcohol provides, and how these may differ across socio-demographics. It is also critical to reflect on why consumption patterns in this population is higher than other age groups—perhaps a reflection of the changes that occur in middle age (e.g. children leaving home, retirement, financial position, among others). A greater understanding of the determinants of alcohol consumption, and how they differ across women in this age bracket, is necessary before risk communication strategies are developed. Importantly, messaging regarding reduction needs to occur within a non-competing policy environment, which is likely the largest challenge health promotors will face. Finally, further research might also look at risk perception of breast cancer diagnoses as our data lead us to question whether medical advances in the detection and treatment of breast cancer may have actually led to poorer adherence to preventative measures.

## Supporting information

S1 Table Interview guide. (PDF)

S2 Table Completed COREQ checklist. (PDF)
