# Supplementary material for: Alcohol and breast cancer risk: Middle-aged women’s logic and recommendations for reducing consumption in Australia
Source: PLoS One. 2019 Feb 13;14(2):e0211293. doi: 10.1371/journal.pone.0211293 (PMC6374008; doi:10.1371/journal.pone.0211293)
Supplement: S1 Table — (PDF) [file pone.0211293.s001.pdf]

S1 Table: Interview Guide

Alcohol and Breast Cancer Risk: Interview Schedule

**Introductory script**
Thanks for expressing interest in this project. It seems to be something that's really connected with people. As you saw in the ad, we're interested in talking to women about what they know about breast cancer and where they've got this information. We're also interested in how alcohol is or isn't a part of the lives of women in this age group.<br><br>I'm going to explain a bit about the interview first to make sure you know what you're getting into, then if you agree to participate we can sign the consent form and get started.<br><br>Throughout the interview if I ask anything that you'd prefer not to answer, there's no issue with that you can just say 'I'd rather not discuss that', and we'll move on. If you'd like to stop the recording or interview at any time please just let me know.<br><br>There's no right or wrong answers and we don't have any expected answers. We're interested in what women in this age group know and think; their opinions and experiences. So in that respect you're already well-qualified! We really appreciate your time today because we think hearing from women like you will help us develop helpful ways of engaging with breast cancer at a population level.<br><br>I am going to audio-record this interview, and we're going to send off the voice recording to get typed-up. When it's typed up your details will be removed from the transcript, so the information won't be linked to you anymore. In terms of what we're going to do with what you tell us; we're just going to look through the interviews to compare and contrast what the women have told us throughout the different interviews to see if there are common themes. Did you have any questions before I ask you to sign the consent form? The consent form is just a document to show that you have got a good understanding of what will happen in the interview and you're still willing to participate.
**Interview Questions**
Keeping healthy and preventing disease
Really broadly, this study is looking into what kind of things people do or don't do to stay healthy, and/or to prevent disease. Can you tell me about anything you do to stop yourself from developing illness?
Are some of these things easier to do than others? Why do you think that is?
All the research evidence tells us that drinking is most common in the age group of middle-aged women 45-64 – so your age group. What are your thoughts on why this is the case?
Are any of these similar to the reasons that you drink alcohol? Are there any differences?
So that's the 'why' – what's the 'when, where, what, how, with whom'? Tell me about some of these things.
If you tend to drink in your home, can you also tell me a little bit about where you tend to get your alcohol from, and why?
Have there been times in your life where you've wanted to reduce your alcohol consumption for some reason? What was that like?
What helped throughout that process or what was hard?
There are lots of different attitudes and opinions about how much alcohol is safe and how much isn't. Do you have a strong opinion on this?
If the government or another health organisation wanted to do something about that 'jump' in rates of alcohol consumption for women your age, what should they do?
Do you think that would be successful?
What do you think might be things that would make it hard for women to reduce their alcohol consumption if they wanted to?
Is there anything that could help? Any strategies, etc?

S1 Table: Interview Guide

Understanding breast cancer specifically
Ok, so that's a bit about alcohol as one of the things people might do to prevent disease. Now we'll switch track a bit and talk about breast cancer. Have you spent much time thinking about why some women get breast cancer and why some don't?
What are your views on this?
Has breast cancer been 'on the radar' for you, or have you been more concerned about other illnesses? Or perhaps you'd prefer not to think about any of them?
Do any of the healthy life things we talked about before apply to breast cancer? Can anything be done to reduce your chances of breast cancer?
You might have already heard about a mammogram. This is part of the early risk identification process for breast cancer. What are your views on having one of these?
What is your opinion on what can be done for those that get breast cancer?
Why did you want to volunteer for the study? Was there something about the ad that you found interesting?
How do you understand the link between alcohol and breast cancer?
Health information and trust
Where do you think you've learnt the most about breast cancer over the course of your life?
Of all the places you could get information, which would you be most likely to believe? How do you decide which information can be trusted?
Have you ever questioned information you've heard about breast cancer? Can you give me an example?
Linking breast cancer and alcohol
If you knew that the way you consume alcohol now was categorically, definitely going to increase your chances of having breast cancer, would you do anything differently? Can you tell me about it?
Something we're interested in is whether knowing more about the links between alcohol and breast cancer would change the way that you felt about drinking. What are your thoughts on this?
What's your view on how much attention should be paid to all the risks/the risks about breast cancer?
Finishing up
I think that's all the questions I'd like to ask today. Is there anything you wanted to add before I turn off the tape recorder? Were there any questions you thought I'd ask that I haven't?
